# Evaluation of rheumatologists’ knowledge of biosimilars

**DOI:** 10.1186/s42358-025-00465-4

**Published:** 2025-07-17

**Authors:** Thayane Furtado Rolim Lima, Lia Poti Gomes Cordeiro, Kirla Wagner Poti Gomes, Carlos Ewerton Maia Rodrigues

**Affiliations:** 1 Resident in Rheumatology, General Hospital of Fortaleza (HGF), Fortaleza, Brazil; 2 Resident in Internal Medicine, Waldemar Alcântara General Hospital (HGWA), Fortaleza, Brazil; 3 Health Sciences from University of Fortaleza (Unifor), General Hospital of Fortaleza (HGF), Fortaleza, Brazil; 4 Graduate Program in Medical Sciences, Universidade de Fortaleza (Unifor), Fortaleza, Brazil; 5 Universidade Federal do Ceara, Fortaleza, Brazil

**Keywords:** Biosimilars, Biosimilar drugs, Interchangeability, Extrapolation

## Abstract

**Introduction:**

Biosimilars reduce the cost of biologic therapy without compromising safety and effectiveness. In this study we evaluated Brazilian rheumatologists’ knowledge and perceptions of biosimilars.

**Methods:**

Cross-sectional and descriptive study based on a questionnaire containing 17 items on familiarity, knowledge and perceptions of biosimilars.

**Results:**

Answers were received from 135 rheumatologists, of whom 97.8% were familiar with biosimilars and 92.5% had at some time prescribed them, but only 47.7% felt comfortable prescribing them to stable patients and 62.2% strongly disagreed with automatic substitution. In addition, 51.9% preferred naive patients when starting treatment with biosimilars.

**Conclusion:**

Despite the growing acceptance of biosimilars, many physicians remain reluctant. Evidence-based continuing education is essential to clarify these issues.

## Introduction

The advent of biologic drugs radically changed the treatment of inflammatory conditions and is now a core component of immune-mediated rheumatological therapy [1], although its high cost poses a significant budgetary challenge to the health systems [2, 3]. In Brazil, approximately 70% of the population depends exclusively on the public healthcare system, the Unified Health System (SUS), and biologic therapies account for nearly 50% of the government’s annual pharmaceutical expenditures [4, 5]. In 2022, an estimated 60,000 SUS patients were treated with adalimumab [6]. As a cost-effective alternative, biosimilars are now available on the market, offering a safety and efficacy profile equivalent to that of their reference biologics.

Since 2016, ANVISA, the Brazilian health regulatory agency, has approved biosimilars for several biologic agents commonly used in rheumatology, including adalimumab, infliximab, etanercept, and rituximab. In Brazil, biosimilars are regulated by ANVISA through RDC 55/2010, which established the initial framework for demonstrating comparability in quality, efficacy, and safety. In the SUS, prescribing follows the National Clinical Protocols and Therapeutic Guidelines (PCDT), which include biosimilars, and switching of originator biologics with biosimilars has been implemented as part of national policy. Over 50 biosimilars have been approved to date, expanding therapeutic options nationwide [7].

In addition, public-private partnerships help reduce costs and boost local production through technology transfers [4]. A recent experience from a Brazilian private health provider illustrates the low penetration of biosimilars and their cost-saving potential. Between January and August 2023, a total of 547 patients receiving care for autoimmune diseases at the operator’s infusion center were analyzed. Of these, 81.8% were treated with originator biologics, 11.2% received originators for which a biosimilar exists, and 5.6% were being treated with biosimilars [8]. As observed in Poland, this scenario may reflect the market dynamics in which biosimilars, despite their cost-effectiveness and comparable efficacy, face competition not only from originator biologics but also from newer reference products that do not yet have biosimilar alternatives. As a result, the overall adoption of biosimilars may be underestimated, not due to lack of confidence or regulatory barriers, but rather due to the expanding availability of novel biologic therapies that are not subject to biosimilar [9].

Following structured substitution strategies, including clinician education and protocol-based switching, the provider achieved a 55.9% reduction in treatment costs among 44 patients from September to December 2023 [8].

However, a proper understanding among physicians of the regulation, pharmacovigilance, interchangeability and indication extrapolation of biosimilars is necessary before these drugs can become mainstay in therapy [10].

Some authors have detected gaps in rheumatologists’ knowledge of biosimilars [11, 12], stressing the need for additional training to subsidize clinical decision making and strengthen trust [13]. In this study, we evaluated the knowledge, acceptance, familiarity and perception of these therapies in a sample of Brazilian rheumatologists.

## Methods

This descriptive, cross-sectional study was conducted at the General Hospital of Fortaleza (HGF) between January and February 2025. The sample consisted of Brazilian practicing rheumatologists who participated voluntarily. Information on age, sex, professional background and knowledge and perception of biosimilars was collected using an online questionnaire with 17 multiple choice items (Appendix A). The participants were Brazilian rheumatologists recruited via professional networks, email, and social media platforms such as WhatsApp and Instagram. Participation was voluntary and included both affiliated and non-affiliated rheumatologists. The questionnaire was developed by the authors based on a comprehensive review of the literature and was validated by two experts in the field of rheumatology.

The answers were entered on Excel spreadsheets and categorized according to (i) participant profile, (ii) knowledge of biosimilars, (iii) extrapolation, interchangeability and automatic substitution, and (iv) beliefs and attitudes regarding the use of biosimilars. The collected data were used to make bar and pie charts. The participants gave their informed written consent and the study protocol, compliant with directive 466/2012 of the National Health Board, was approved by the research ethics committee of the HGF (entry #7.348.890).

## Results

### Participant profile

**Fig. 1:**
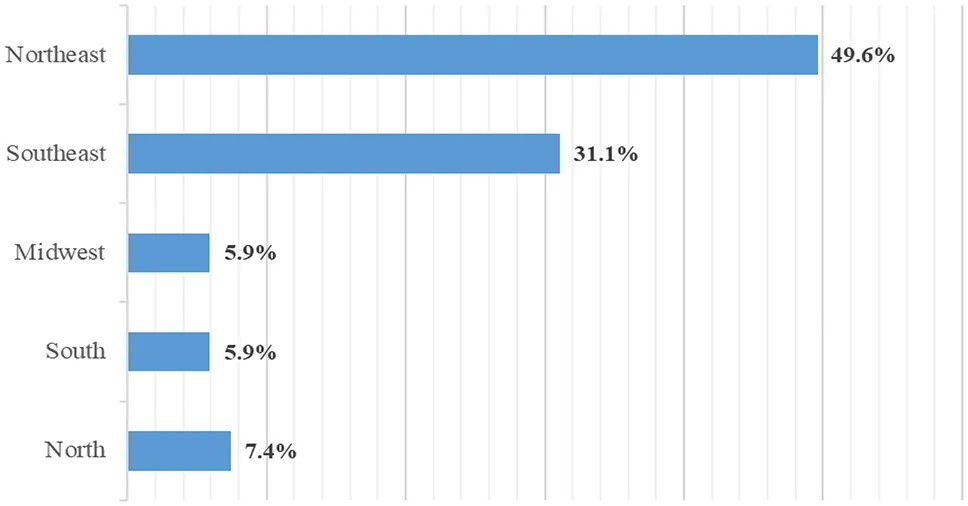
Distribution of the participating rheumatologists across the country’s five main regions

The questionnaire was answered by 135 rheumatologists, most of whom were aged 30–59 years (75.9%). Most were women (64.8%) and most had over 10 years of experience in the field (70.2%). All the geographical regions of the country were represented in the sample, but the Northeast was predominant (49.3%) (Fig. 1). Moreover, 64.1% reported working in both the public and private sector.

### Knowledge of and familiarity with biosimilars

**Fig. 2:**
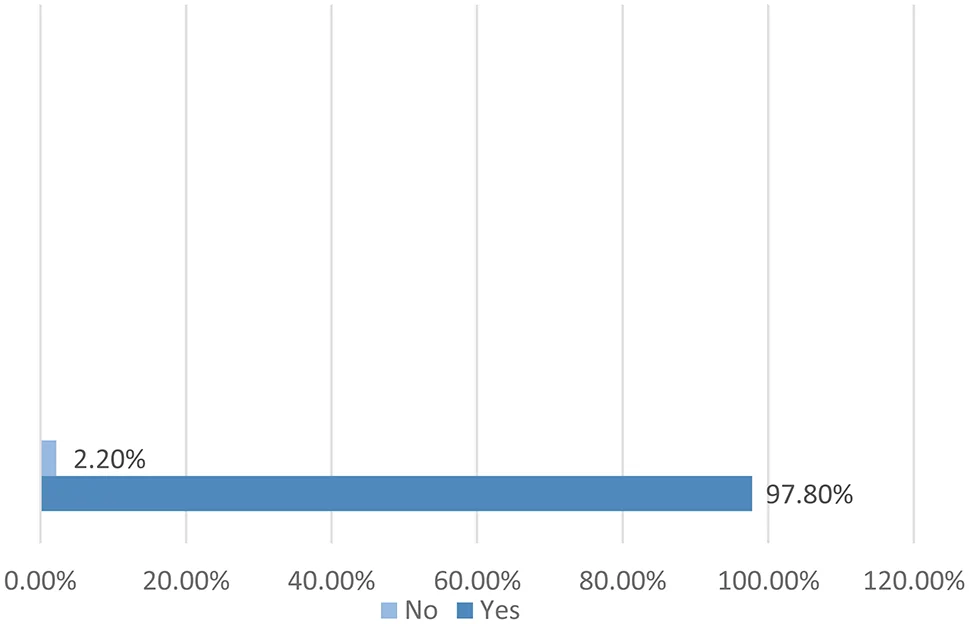
Proportion of participating rheumatologists reporting to be familiar with biosimilars

Almost all the respondents (97.8%) were familiar with the concept of biosimilars (Fig. 2), and 91.8% were aware that equivalence in safety and effectiveness is required for approval. Also, 92.5% had already prescribed biosimilars for rheumatological diseases, and 73.1% said they kept abreast with studies and reviews on the topic.

### Extrapolation, interchangeability and automatic substitution

**Fig. 3:**
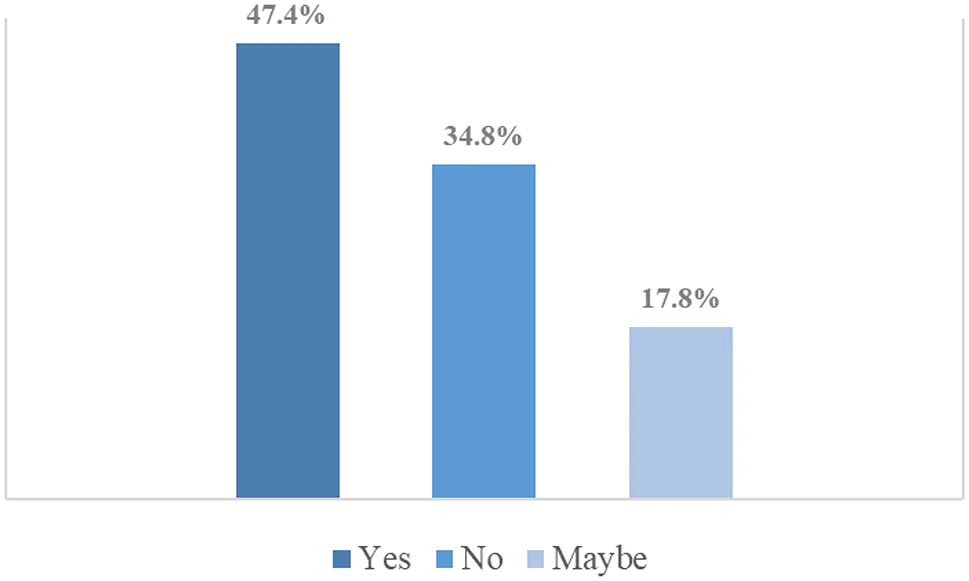
Proportion of participating rheumatologists reporting to have prescribed an approved biosimilar to a stable patient as a replacement for a biologic drug

**Fig. 4:**
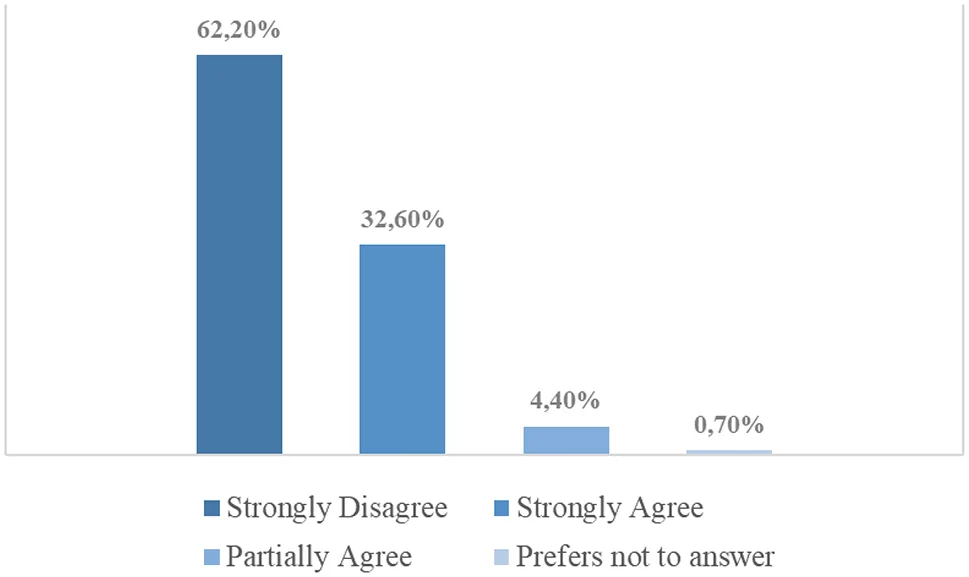
Agreement among the participating rheumatologists regarding automatic substitution

When asked about extrapolation of indications, 89.6% did not think additional clinical trials to test other indications were necessary, while 6.7% believed them to be essential. Nevertheless, 23.1% had an unfavorable opinion of the effectiveness of biosimilars for all indications, while 32.1% were unsure. Nearly half (47.4%) felt comfortable replacing a reference drug with a biosimilar in the treatment of a stable patient, but 34.8% felt uncomfortable and 17.8% expressed doubt (Fig. 3). Finally, 62.2% were strongly against automatic substitution, 32.6% were partially against it, and 4.4% agreed completely. The remaining 0.7% declined to answer (Fig. 4).

### Beliefs and attitudes regarding the prescription of biosimilars

The prescription of biosimilars was predominantly explained by adherence to guidelines and technical directives (41.8%), followed by clinical comparability data and cost reduction (28.1% each). Only 1.5% considered the patient’s opinion a decisive factor.

**Fig. 5:**
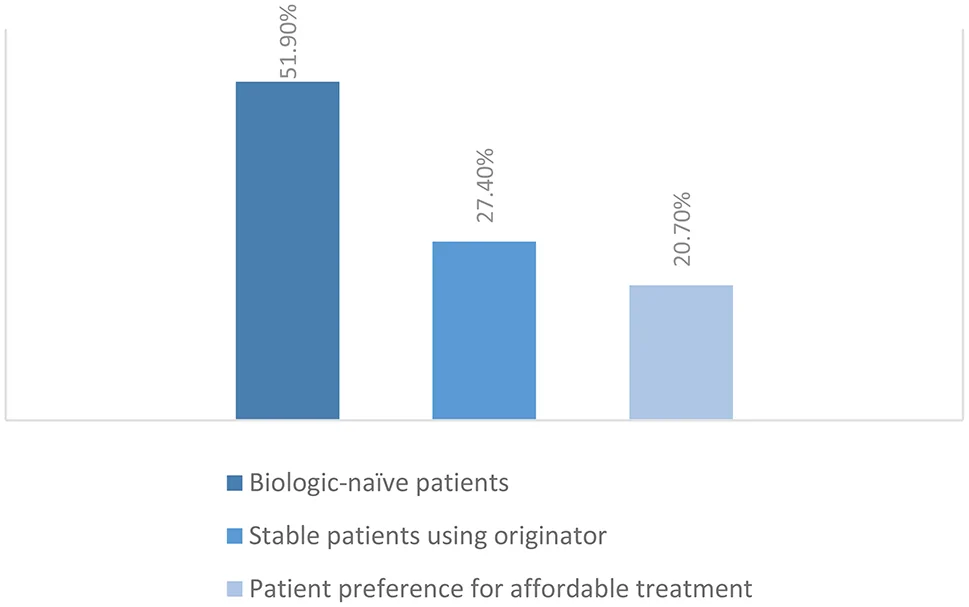
Patient profiles to which the participating rheumatologists preferably prescribed biosimilars

Just over half the participants (51.5%) preferred to prescribe biosimilars to patients with no history of biologic therapy (Fig. 5). The majority (85.8%) believed the prevalence of adverse effects to be similar for biosimilars and their reference drugs, but 68.6% did not trust or were not sure that switching between biosimilars of the same reference drug is safe and effective.

Finally, 84.3% believed that a wider adoption of biosimilars can facilitate access to biologic therapy.

## Discussion

This is to our knowledge the first Brazilian study in the last decade to evaluate rheumatologists’ knowledge, beliefs and perceptions of biosimilars. Our results provide a comprehensive picture of the acceptance of biosimilars and the associated challenges. Most of the respondents were women aged 30–59 years, matching the demographics of the Brazilian Society of Rheumatology [14]. Predominantly located in the Northeast, many of the respondents worked in both the public and private sector, a fact that may have influenced their view on the prescription of biosimilars.

Almost all were familiar with the definition of biosimilars and adhered to guidelines. In comparison, in another study 83% of the respondents were familiar with the definition given by the Food and Drug Administration (FDA) [11]. Most were aware of the requirement of equivalence between biosimilars and their reference drugs with regard to effectiveness and safety, contrasting with another study in which only half the participants were aware of such criteria [12]. In another Brazilian survey performed in 2010, Brazilian rheumatologists’ knowledge of biosimilars proved to be limited. Although 67% claimed to be familiar with them, only 34% correctly defined the term [15]. Moreover, the rheumatologists in our sample reportedly keep abreast with the literature on the topic, suggesting a strong interest in and endorsement of the concept.

However, despite the high level of knowledge and familiarity observed, over half the respondents did not feel comfortable replacing the reference drug with a biosimilar. In contrast, 91% of the participants in another study were in favor of substitution [12]. Data from a rheumatologists’ opinions study showed that the main concern regarding commercialization was pharmacovigilance [15]. Thus, our results point to the need for Brazilian rheumatologists to engage in continuing education [16]. 

In addition, only half the participants believed biosimilars to be as safe and effective as their reference drugs for all indications, despite being aware of the concept of extrapolation of indications. In another study, only 12% felt comfortable prescribing biosimilars [12]. 

The idea of switching between biosimilars based on the same reference drug, without consulting an expert, was met with considerable resistance, indicating a limited understanding of the concept of interchangeability, according to which one biosimilar can be replaced with another biosimilar without compromising effectiveness or safety [13]. 

International regulatory agencies guarantee the safety of such substitutions [17, 18], and the Brazilian regulatory agency established the criteria for interchangeability in 2017 [19]. Although an authorization is not required, informing the original prescriber and involving the patient is a recommended practice [20, 21]. Multiple changes within a year should be avoided since the persistence of the treatment in this period is similar to that of the respective reference drug [22, 23]. 

Patients with no history of biologic drug use were the preferred profile for prescription, followed by stable patients, matching the findings of another study [11]. This approach is consistent with the recommendation of prescription of biosimilars to stable or naive patients [24]. 

Adherence to guidelines was the decisive factor for prescribing biosimilars in our sample. Only 28.1% laid more emphasis on cost reduction. In another studies, however, more than half of the rheumatologists specified cost reduction as the main reason for prescribing biosimilars [15, 25], highlighting the need to clarify these issues among prescribers.

Interestingly, while 84.3% of respondents acknowledged that biosimilars improve access to biologic therapy, only 28.1% cited cost reduction as a primary reason for prescribing them. This apparent contradiction underscores a disconnect between the perceived systemic benefits of biosimilars and their practical implications in clinical decision-making. Although lower cost is the principal advantage of biosimilars from a policy and budgetary perspective, individual prescribers may not directly observe or be impacted by these savings, particularly within SUS, where drug procurement is centralized and decisions are often made through institutional bidding processes.

Consequently, physicians may associate improved access more with policy-level strategies and broader availability than with the direct economic incentives that drive biosimilar adoption in other healthcare systems. This highlights the importance of aligning clinical education with the broader economic rationale behind biosimilar integration, to foster more coherent adoption strategies.

International experiences highlight that the successful adoption of biosimilars depends largely on national policy and system-level coordination. In Spain, biosimilars generated €2.3 billion in savings over a decade, mainly through competitive pricing and hospital procurement strategies [26]. Norway and the UK also demonstrated that structured approaches, such as national tendering, prescriber incentives, and switching protocols—can expand access to biologics while reducing costs [27]. In Poland, real-world data showed that biosimilar policies doubled treatment access without increasing healthcare spending, and enhanced disease control when combined with physician oversight and continuous education [8, 28]. These findings reinforce that effective biosimilar integration requires more than prescriber willingness, it demands supportive health system frameworks.

Our study was limited by the cross-sectional design which made it impossible to evaluate changes in acceptance over time. Also, the self-administered questionnaire may have influenced the participants’ perceptions, and although the demographic profile of respondents resembles national data, the use of spontaneous recruitment via social media, email, and WhatsApp may have introduced a selection bias, with a higher proportion of respondents from the Northeast, despite the greater concentration of rheumatologists in the Southeast. This geographic imbalance may limit the generalizability of the results. Finally, institutional, economic and regulatory issues were not analyzed in depth, limiting our understanding of the hurdles preventing the adoption of biosimilars in Brazilian clinical practice.

## Conclusion

Our study shows that in general Brazilian rheumatologists have knowledge of biosimilars and often prescribe them. The preference for naive or stable patients to initiate treatment with biosimilars is consistent with international recommendations. The participating rheumatologists also reported keeping up with the literature on the topic and evidence-based recommendations.

Despite doubts and unfavorable perceptions of automatic substitution, our review of the literature suggests rheumatologists’ awareness of the issue has grown over the last decade.

Information on biosimilars should be better disseminated by expanding educational programs and fomenting scientific discussions, thereby increasing trust and acceptance on part of prescribers.

## Appendix A

**Questionnaire on biosimilars**

1. Are you familiar with the concept of biosimilars?

- ( ) Yes ( ) No

2. Have you ever prescribed biosimilars to treat a rheumatological disease?

- ( ) Yes ( ) No

3. According to regulatory agencies, what is required to approve a biosimilar?
  - Clinical effectiveness only
  - Pharmacokinetic and pharmacodynamic similarity, in addition to comparable safety and immunogenicity
  - Lower cost than that of the reference drug
  - Clinical safety only

4. What is extrapolation of indications when referring to biosimilars?
  - Use of the biosimilar for the tested disease only
  - Approval of the biosimilar for all the indications of the reference drug, with no need for new clinical trials in each case
  - Approval of the biosimilar for one indication only
  - Need to conduct new clinical trials for each indication of the reference drug as a condition for approval

5. Do you believe biosimilars are as effective as their reference drug for all indications?

- ( ) Yes ( ) No ( ) Don’t know

6. Are you comfortable replacing a reference drug with an approved biosimilar in a stable patient?

- ( ) Yes ( ) No ( ) Maybe

7. What is the main decisive factor for you to replace a reference drug with an approved biosimilar?

- ( ) Clinical comparability data
- ( ) The patient’s preference
- ( ) Health care guidelines and consensus
- ( ) Cost reduction

8. In your opinion, can the use of biosimilars expand access to biologic therapy?

- ( ) Yes ( ) No

9. What do you think of automatically substituting a biosimilar for a reference drug, without the consent of the prescribing physician?

- ( ) Strongly agree
- ( ) Partially agree
- ( ) Strongly disagree
- ( ) Decline to answer

10. Do you believe that switching between different biosimilars derived from the same reference drug is safe and efficient?

- ( ) Yes ( ) No

11. Do you think biosimilars have greater, smaller or the same risk of adverse events as the respective reference drug?

- a) Greater  b) Smaller  c) The same

12. What is the most important patient profile when prescribing a biosimilar?

- ( ) Patients with no history of biosimilar use
- ( ) Stable patients using a reference drug
- ( ) Patients preferring less costly treatment

13. Do you keep up with studies and clinical reviews on the safety and effectiveness of biosimilars in you clinical practice?

- ( ) Yes ( ) No

14. What gender do you identify as?

- ( ) Male ( ) Female ( ) Decline to answer

15. Do you work in the public or private sector, or in both?

- ( ) Public ( ) Private ( ) Both

16. How old are you?

- ( ) < 30 years ( ) 40–49 years ( ) 50–59 years ( ) ≥ 60 years

17. How long have you worked as a rheumatologist?

- ( ) < 5 years
- ( ) 5–10 years
- ( ) 11–20 years
- ( ) > 20 years

## Data Availability

No datasets were generated or analysed during the current study.
